# Cannabidiol and Minor Phytocannabinoids: A Preliminary Study to Assess Their Anti-Melanoma, Anti-Melanogenic, and Anti-Tyrosinase Properties

**DOI:** 10.3390/ph16050648

**Published:** 2023-04-26

**Authors:** Katarzyna Gaweł-Bęben, Karolina Czech, Simon Vlad Luca

**Affiliations:** 1 Department of Cosmetology, University of Information Technology and Management in Rzeszów, 35-225 Rzeszów, Poland; 2 Biothermodynamics, TUM School of Life Sciences, Technical University of Munich, 85354 Freising, Germany

**Keywords:** cannabigerol, cannabichromene, cannabinol, melanin release, B16F10 cells, malignant melanoma cells, tyrosinase

## Abstract

Currently, there is an increased interest from both scientists and consumers in the application of cannabis/hemp/phytocannabinoids in skin-related disorders. However, most previous investigations assessed the pharmacological properties of hemp extracts, cannabidiol (CBD), or tetrahydrocannabinol (THC), with very few studies focusing on minor phytocannabinoids from hemp. In this context, the current work explored the *in vitro* anti-melanoma, anti-melanogenic, and anti-tyrosinase effects of cannabidiol (CBD) and three minor phytocannabinoids, namely cannabigerol (CBG), cannabinol (CBN), and cannabichromene (CBC). Among the tested human malignant melanoma cells (A375, SH4, and G361), only A375 cells were highly susceptible to the 48 h treatment with the four phytocannabinoids (IC_50_ values between 12.02 and 25.13 μg/mL). When melanogenesis was induced in murine melanoma B16F10 cells by α-melanocyte stimulating hormone (αMSH), CBD, CBG, and CBN significantly decreased the extracellular (29.76–45.14% of αMSH+ cells) and intracellular (60.59–67.87% of αMSH+ cells) melanin content at 5 μg/mL. Lastly, CBN (50–200 μg/mL) inhibited both mushroom and murine tyrosinase, whereas CBG (50–200 μg/mL) and CBC (100–200 μg/mL) down-regulated only the mushroom tyrosinase activity; in contrast, CBD was practically inactive. The current data show that tyrosinase inhibition might not be responsible for reducing the melanin biosynthesis in α-MSH-treated B16F10 cells. By evaluating for the first time the preliminary anti-melanoma, anti-melanogenic, and anti-tyrosinase properties of CBN and CBC and confirming similar effects for CBD and CBG, this study can expand the utilization of CBD and, in particular, of minor phytocannabinoids to novel cosmeceutical products for skin care.

## 1. Introduction

With the discovery of the endocannabinoid system in the skin and its involvement in maintaining skin homeostasis, the interest of both scientists and consumers in the application of cannabis/hemp extracts or phytocannabinoids in the treatment of skin-related disorders has increased. Synthesized by hair follicles, epidermal cells, and sebaceous glands, endocannabinoids in the skin may modulate cannabinoid receptors type 1 and 2 (CB_1_ and CB_2_) and transient receptor potential vanilloid-1 (TRPV_1_); almost all skin cell types express these receptors. Dysregulation of the endocannabinoid signaling may lead to eczema, psoriasis, pigmentation disorders, atopic dermatitis, or impaired hair growth [1,2]. The growing number of scientific publications show that cannabis, hemp, or cannabinoids (endocannabinoids, phytocannabinoids, synthetic cannabinoids) may have beneficial effects in ameliorating pruritus [3,4,5], atopic dermatitis [6], psoriasis [7,8], acne [9,10], and eczema [11,12]. Among phytocannabinoids, cannabidiol (CBD) might be suitable for such applications, as it possesses additional anti-inflammatory, analgesic, moisturizing, and anti-wrinkle properties [13]. The use of phytocannabinoids in treating skin problems is also supported by their topical application since a better control of the active substance concentration at the site of action is expected; thus, the risk of side effects that might occur upon oral administration is reduced [14]. Non-cancer skin diseases (e.g., hyper-/hypo-pigmentation disorders linked to impaired melanin biosynthesis and tyrosinase activity), as well as malignant pathologies (e.g., melanoma), constitute promising research directions with cannabinoids, as recently reviewed by several authors [2,15,16,17]. 

Melanocytes are skin cells that produce endocannabinoids (anandamide, AEA; 2-arachidonoylglycerol) and their target receptors (CB_1_, CB_2_, TRPV_1_). At high concentrations, AEA was shown to cause melanocyte apoptosis, whereas, at low concentrations, AEA induces melanogenesis and activates tyrosinase. Tyrosinase converts L-tyrosine into L-3,4-dihydroxyphenylalanine (L-DOPA) and, subsequently, into dopaquinone. Thus, tyrosinase is considered the main enzyme involved in melanin synthesis. The mechanism of AEA-induced melanogenesis seems to differ from the most common signaling pathways induced by α-melanocyte stimulating hormone (αMSH) [18]. Due to the undeniable role of endocannabinoids in the homeostasis of melanocytes, the use of phytocannabinoids in treating malignant melanoma and pigmentation disorders is of particular interest.

Around 80% of skin cancer-related deaths worldwide are associated with melanoma, a highly metastatic skin cancer whose incidence continues to rise globally [19]. The primary chemotherapeutics for melanoma treatment are combinations of 5-fluorouracil (5FU) and cisplatin; these agents selectively promote apoptosis in actively dividing cells by interfering with DNA synthesis. Selective inhibitors of v-Raf murine sarcoma viral oncogene homolog B (BRAF), such as temozolomide and dacarbazine, and antibodies targeting T-lymphocyte-associated protein (CTLA4) have also been used in the modern melanoma therapies [20]. Unfortunately, due to the highly metastatic potential and multiple resistance mechanisms of melanoma cells, the mortality rate of this disease remains very high [21]. Therefore, developing novel therapeutic strategies to treat malignant melanoma is critical. 

Numerous researchers investigated the anti-proliferative and pro-apoptotic effects of endocannabinoids, phytocannabinoids, and synthetic cannabinoids in human and murine melanoma cells [22,23]. Additionally, the data from the *in vivo* studies confirmed that phytocannabinoids, alone or in combination, can decrease tumor growth and promote autophagy and apoptosis in different melanoma models [24]. Most data related to the anti-melanoma activity of phytocannabinoids come from experiments based on cannabis/hemp extracts, hemp oils, CBD, or tetrahydrocannabinol (THC). Except for one study showing the anti-proliferative potential of cannabigerol (CBG) in mouse skin melanoma cells [25], the influence of other phytocannabinoids has not been previously investigated *in vitro* or *in vivo*. Concerning the pigmentation-regulating properties of cannabis/hemp extracts or phytocannabinoids, the results are sometimes contradictory. Essential oils, aqueous, and methanol extracts obtained from hemp flowers downregulated melanin biosynthesis and inhibited tyrosinase activity [26,27,28], whereas THC has been shown to inhibit melanin synthesis in human hair follicles [29]. Furthermore, CBD and THC significantly increased tyrosinase activity and melanin content in murine melanocytes [30,31]. However, the role of other phytocannabinoids in regulating melanogenesis has not been described. 

**Figure 1:**
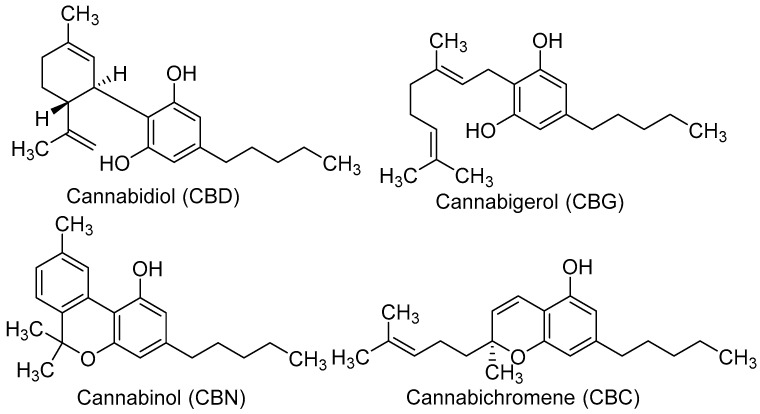
Structure of the phytocannabinoids included in the current study.

Considering the recently increased worldwide interest in cannabis/hemp/phytocannabinoids-based cosmetic products, there is an imperious need to bring solid scientific evidence to confirm their safety profile and beneficial effects on the skin. Furthermore, most studies that explored the skin-related effects of phytocannabinoids are generally limited to CBD or THC. Thus, this work aimed to assess for the first time the *in vitro* anti-melanoma, anti-melanogenic, and anti-tyrosinase properties of three minor phytocannabinoids from hemp, namely cannabigerol (CBG), cannabinol (CBN), and cannabichromene (CBC) (Figure 1). The influence of the selected phytocannabinoids on the viability of human malignant melanoma A375, SH4, and G361 cells was evaluated by the neutral red uptake assay. The modulation of melanin biosynthesis was assessed in αMSH-stimulated murine melanoma B16F10 cells by quantifying the extracellular and intracellular melanin content. Lastly, the effects on tyrosinase activity were studied in both mushroom and murine tyrosinase. 

## 2. Results and Discussion

### 2.1. Influence of Phytocannabinoids on Malignant Melanoma Cell Viability

**Figure 2:**
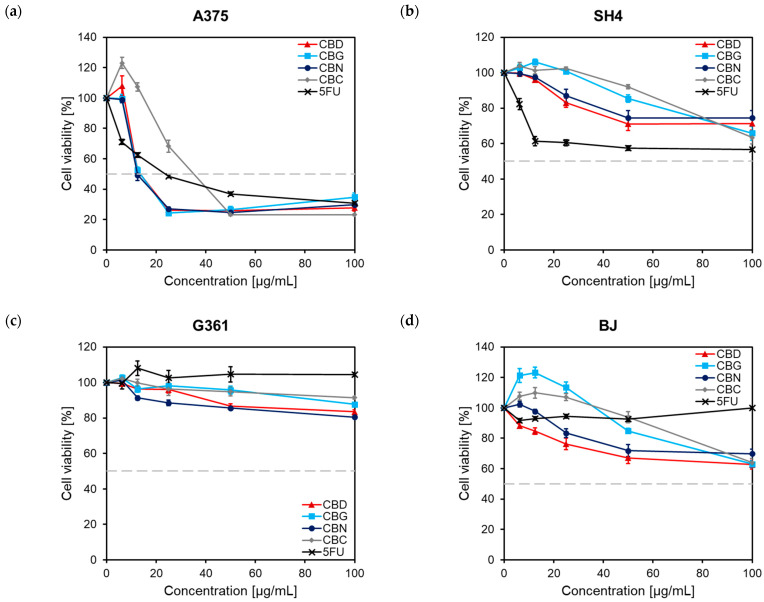
Concentration-response curves showing the effect of phytocannabinoids (6.25–100 μg/mL) on the viability of (**a**) A375 cells, (**b**) SH4 cells, (**c**) G361 cells, and (**d**) BJ fibroblasts. Cells were plated in 96-well plates (3 × 10^3^/well) and incubated for 48 h with different concentrations of phytocannabinoids (10–200 μg/mL). Graphs present the percentage cell viability (%) compared to the control cells, set at 100%. Each point illustrates the average ± S.E.M. of at least three experiments performed in triplicate. The gray-dashed horizontal line indicates the viability of 50%; 5FU, 5-fluorouracil, CBC, cannabichromene; CBD, cannabidiol; CBG, cannabigerol; CBN, cannabinol.

The influence of four phytocannabinoids (CBD, CBG, CBN, and CBC) previously isolated from hemp flower extracts [32,33] on the viability of several human malignant melanomas (A375, SH-4, and G361) cells was initially studied. The results, presented as concentration-response curves (Figure 2), show that the tested compounds (6.25–100 μg/mL) were significantly cytotoxic to A375 cells but not to SH4 and G361 cells; in addition, the four phytocannabinoids did not significantly affect the viability of non-cancer skin BJ fibroblasts. Based on the calculated IC_50_ values (Table 1), it can be observed that CBN and CBC displayed similar cytotoxicity as 5FU (IC_50_ between 23–30 µg/mL), whereas CBD and CBG were twice as cytotoxic (IC_50_~12 µg/mL). 

In a previous study, CBD reduced the viability and proliferation of malignant melanoma (A375, FM55P, SK-MEL-28, and FM55M2) cells in a concentration-dependent manner; the IC_50_ values in these cell lines were between 3.81 and 7.75 µg/mL. In addition, the viability of human immortalized HaCaT keratinocytes was not significantly affected [34]. The lack of CBD toxicity towards non-cancer skin cells also agrees with the research of Vacek et al. [35], when no reduction in the viability of HaCaT keratinocytes and normal human dermal fibroblasts was observed over the concentration range of 0.78–100 μM. Burch et al. [36] showed that CBD displayed significant cytotoxic effects in murine melanoma B16 cells at concentrations between 40 and 200 μg/mL. Choi et al. [37] also evaluated the effects of CBD in A549 cells, demonstrating that CBD exhibited a time- and concentration-dependent cytotoxicity when applied at concentrations ranging from 5 to 80 μM in a time course ranging from 6 to 36 h.

The anti-melanoma activity of other endogenous, natural, or synthetic cannabinoids is also documented. For instance, the endocannabinoid AEA exerted cytotoxic effects in A375 cells via modulation of caspase-dependent apoptotic signaling pathways [38], in the study of Baek et al. [25], CBG displayed an IC_50_ value of 31.31 µg/mL in mouse skin melanoma cells, comparable to the one reported in the current study for A375 cells. Additionally, THC (1 μM) and a synthetic cannabinoid WIN-55,212-2 (100 nM) inhibited the growth of melanoma A375 and B16 cells but not of normal melanocytes [39]. However, to the author’s knowledge, the anti-melanoma effects of CBN and CBC are presented herein for the first time.

### 2.2. Influence of Phytocannabinoids on Melanin Synthesis 

**Figure 3:**
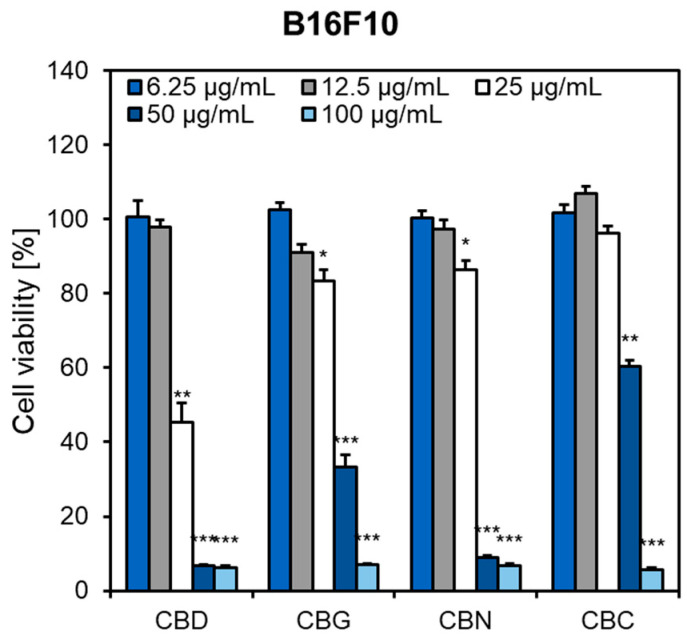
Influence of phytocannabinoids (6.25–100 μg/mL) on the viability of B16F10 cells. Cells were plated in 96-well plates (3 × 10^3^/well) and incubated for 48 h with different concentrations of cannabinoids. Bars present the percentage cell viability (%) compared to the control cells, set at 100%. Each bar illustrates the average ± S.E.M. of at least three experiments performed in triplicate. * *p* < 0.05; ** *p* < 0.01; *** *p* < 0.001 vs. control. CBC, cannabichromene; CBD, cannabidiol; CBG, cannabigerol; CBN, cannabinol.

The influence of phytocannabinoids (6.25–100 µg/mL) on melanin synthesis was investigated in a murine melanoma B16F10 cell model. Considering the previously reported cytotoxic effects of some endocannabinoids in B16 mouse melanoma cells [40], the cytotoxicity of CBD, CBG, CBN, and CBC was initially assessed (Figure 3). The highest cytotoxic effects were detected for CBD, which reduced the percentage of viable cells by ca. 50% at 25 µg/mL. In contrast, CBC showed the lowest cytotoxicity at the same concentration. Based on the data presented in Figure 3, concentrations of 2.5 and 5 µg/mL were considered non-toxic (safe) and used as working concentrations for further experiments.

**Figure 4:**
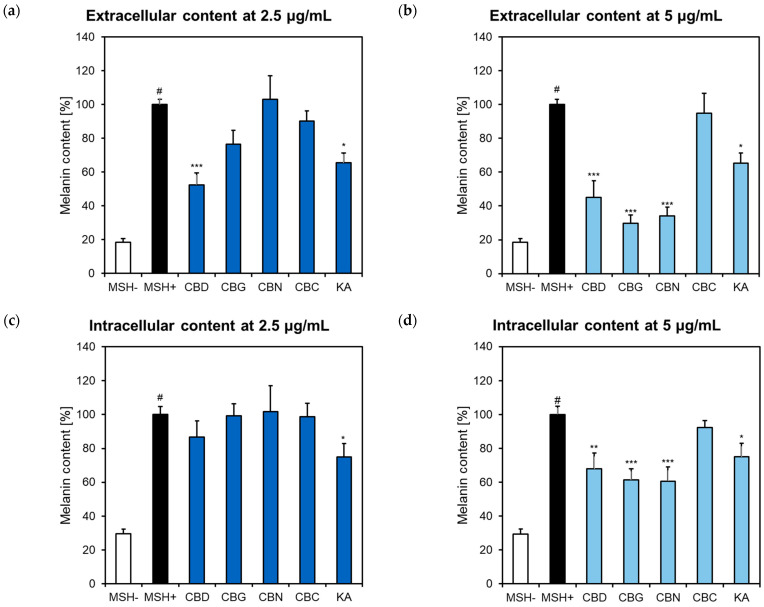
The effect of phytocannabinoids on melanin content in αMSH-stimulated B16F10 cells presented as extracellular content at (**a**) 2.5 μg/mL and 5 μg/mL (**b**), and extracellular content at (**c**) at 2.5 μg/mL and (**d**) 5 μg/mL. B16F10 cells were plated in 6-well plates (0.5 × 10^5^/well) and incubated for 48 h with cannabinoids; αMSH (10 nM) was added to stimulate the melanin production; kojic acid (KA, 200 μg/mL) was a positive control. Graphs presented the percentage of melanin content compared to αMSH stimulated control (αMSH+) cells, set at 100%. Each bar illustrates the average ± S.E.M. of at least three experiments performed in triplicate; # *p* < 0.001 vs. non-stimulated (αMSH-) cells; * *p* < 0.05; ** *p* < 0.01; *** *p* < 0.001 vs. αMSH+ cells. CBC, cannabichromene; CBD, cannabidiol; CBG, cannabigerol; CBN, cannabinol.

The evaluation of melanin release revealed that, at 2.5 μg/mL, only CBD significantly reduced the extracellular content to 52.49% of αMSH-stimulated cells (Figure 4a). However, at 5 μg/mL, CBG and CBN additionally displayed inhibitory effects (29.76% and 34.14% of αMSH-stimulated cells, respectively) (Figure 4b). Concerning the melanin synthesis, CBD, CBG, and CBN significantly decreased the intracellular content, only at 5 μg/mL (67.87%, 61.25%, and 60.59% of αMSH-stimulated cells, respectively) (Figure 4c,d). In contrast, CBC did not show important modulatory effects of the extracellular or intracellular melanin content. 

**Figure 5:**
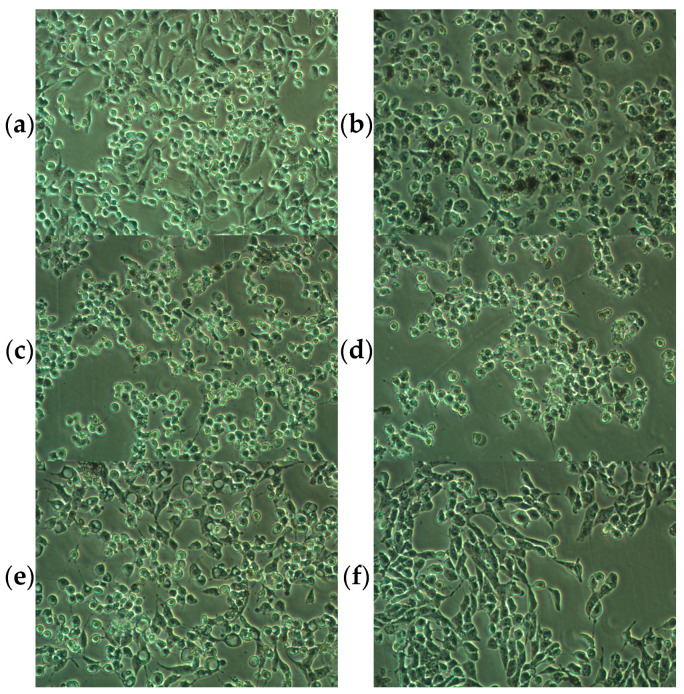
Morphology of murine melanoma B16F10 cells grown for 48 h in the presence of (**a**) DMSO (untreated, αMSH- negative cells), (**b**) αMSH (10 nM, αMSH+ cells), (**c**) αMSH + CBD (5 μg/mL), (**d**) αMSH + CBG (5 μg/mL), (**e**) αMSH + CBN (5 μg/mL), (**f**) αMSH + CBC (5 μg/mL); 10× magnification; pictures are representative for three experiments.

To rule out the possibility that the inhibitory melanin production could have appeared from the reduced cell viability, especially at 5 μg/mL, the microscopic examination of treated cells was performed to complement the results from the previously analyzed neutral red uptake assay. Compared to the positive control (αMSH+) cells, B16F10 melanoma cells treated with αMSH and CBD, CBG, or CBN showed a round shape and less darkly pigmented cells. B16F10 cells co-treated with αMSH and CBC were spindly in shape and seemed darker in color (Figure 5).

The influence of cannabis/hemp/cannabinoids on αMSH-induced melanogenesis has been scarcely explored. For instance, Chen et al. [41] showed that CBD and two synthetic cannabinoids (S-88745 and S-91253) reduced the intracellular melanin content in αMSH-stimulated B16F10 cells at concentrations of 0.0256–0.54 μM. The available data indicate the involvement of phytocannabinoids in regulating basal melanin biosynthesis in various murine and human experimental models. An aqueous hemp flower extract, containing mostly CBD and cannabidiolic acid, displayed a concentration-dependent inhibitory activity on the L-DOPA turnover induced by H_2_O_2_ in mouse skin tissues over the concentration range of 1–500 µg/mL [26,28]. In addition, THC treatment (0.2–2 µM) was also shown to inhibit melanin synthesis in human hair follicles [29].

However, recent data obtained from *in vitro* experiments on primary human melanocytes indicate that phytocannabinoids, namely CBD and THC, induce melanin synthesis. Hwang et al. [30] measured the melanin content and intracellular tyrosinase activity in human epidermal melanocytes treated with CBD. The melanin content and tyrosinase activity were augmented in a concentration-dependent manner (1–6 µM). CBD also increased tyrosinase gene and protein expression levels. Further investigations on the activation of specific signaling molecules revealed that the CBD-induced melanin biosynthesis was mediated by the activation of complement receptor 1 (CR_1_) and p38–Mitogen-activated protein kinase (MAPK) pathway [30].

Additionally, Goenka [31] investigated the influence of THC and CBD on melanogenesis in epidermal melanocytes from neonatal darkly-pigmented and lightly-pigmented human donors. The study showed that a 6-day treatment with both THC and CBD at 1–2 µM increased the intracellular melanin levels and dendrite formation (indication of increased melanosome transfer). Interestingly, the experiments with selective CR_1_ or CR_2_ receptor agonists did not confirm the involvement of CR_1_ in this process [31]. The contradictory data on the phytocannabinoids’ effects on melanogenesis in different experimental models indicate that further studies are still required.

### 2.3. Influence of Phytocannabinoids on Tyrosinase Activity 

One of the mechanisms that might be responsible for a decrease in melanin biosynthesis is related to the inhibition of tyrosinase, a metalloenzyme that influences the first two rate-limiting melanogenesis steps [42]. Previous studies [43,44,45] showed that natural compounds might interact differently with tyrosinase enzymes of various origins [46]. Alignment of the protein sequence of mushroom, murine, and human tyrosinase using BLAST tool [47] showed a 23.23% amino acid identity between mushroom and murine tyrosinase, a 23.42% identity between mushroom and human tyrosinase and an 85.37% identity between murine and human enzymes. Thus, the four phytocannabinoids were evaluated for their tyrosinase inhibitory properties using mushroom tyrosinase from *Agaricus bisporus* and murine tyrosinase from B16F10 murine melanoma cell lysates.

**Figure 6:**
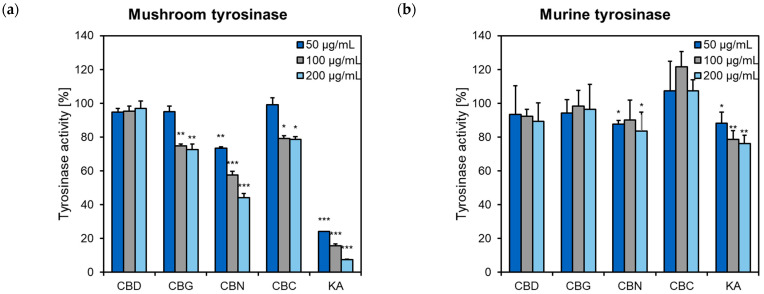
The effect of cannabinoids (50–200 μg/mL) on (**a**) mushroom tyrosinase and (**b**) murine tyrosinase. Data are presented as a percentage of tyrosinase activity compared to solvent (DMSO) control, set at 100%. Kojic acid (KA, 50–200 μg/mL) was a positive control. Each bar illustrates the average ± S.D. of three determinations; * *p* < 0.05; ** *p* < 0.01; *** *p* < 0.001 vs. control. CBC, cannabichromene; CBD, cannabidiol; CBG, cannabigerol; CBN, cannabinol.

CBN, CBC, and CBG showed inhibitory activity towards mushroom tyrosinase at 100 and 200 µg/mL (Figure 6a). Only CBN showed moderate activity in the murine tyrosinase inhibitory assay (Figure 6b). These results indicate that the inhibition of tyrosinase activity might not be responsible for the observed reduction in the melanin release and content in αMSH-treated B16F10 cells (see Section 2.2).

The inhibitory activity of cannabis/hemp/cannabinoids was previously evaluated. For instance, hemp flower methanol extracts inhibited mushroom tyrosinase [27]. The tyrosinase inhibition was also previously observed for essential oils obtained from different hemp varieties (21.31–31.73 mg kojic acid equivalents/g oil) [26]. CBD and two synthetic cannabinoids, S-88745 and S-91253, reduced the tyrosinase activity in αMSH-stimulated B16F10 cells over the concentration domain of 0.0256–0.54 μM [41].

Nonetheless, the influence of minor phytocannabinoids (CBC, CBG, and CBN) on mushroom or murine tyrosinase activity has not been thoroughly described. One patent application indicated the inhibitory activity of CBG at 0.5 mg/mL on mushroom tyrosinase and melanin biosynthesis in B16 mouse melanocytes following 3-day treatment [48]. However, the presented data are the first analysis of the mushroom and murine tyrosinase inhibitory properties of CBC and CBN.

## 3. Materials and Methods

### 3.1. Chemicals

Dulbecco’s phosphate-buffered saline (DPBS), neutral red solution (3.3 g/L in DPBS), Dulbecco’s modified Eagle’s medium (DMEM), Eagle’s minimum essential medium (EMEM), synthetic melanin, glucose, L-3,4-dihydroxyphenylalanine (L-DOPA), murine tyrosinase, kojic acid (≥98.5%) were acquired from Sigma Aldrich/Merck (Darmstadt, Germany). Fetal bovine serum (FBS) was from Pan Biotech (Aidebach, Germany), whereas McCoy’s 5a medium was from LGC Standards (Łomianki, Poland). Ethanol, acetic acid, and sodium hydroxide (NaOH) were purchased from Honeywell (Charlotte, NC, USA). Cannabidiol (CBD, >99.0%), cannabigerol (CBN, >98.0%), cannabinol (CBN, >98.5%), and cannabichromene (CBC, >95.0%) were isolated from hemp flower extracts, as described in [32,33]. 

### 3.2. Cell Lines

Human malignant melanoma A375 (ATCC CRL-1619), SH4 (ATCC CRL-7724), and G361 (ATCC CRL-1424) cells, human BJ fibroblasts (ATCC CRL-2522), and murine melanoma B16F10 cells (ATCC CRL-6475) were purchased from LGC Standards (Łomianki, Poland). A375, SH4, and B16F10 cells were kept in DMEM supplemented with glucose (4.5 g/L) and FBS (10%); G361 cells were grown in McCoy’s 5a Medium with 10% FBS, whereas BJ fibroblasts were grown in EMEM containing FBS (10%). All cell lines were cultured at 37 °C in a humidified atmosphere with 5% CO_2_.

### 3.3. Cell Viability Assay (Neutral Red Uptake)

The influence of the tested phytocannabinoids on the viability of melanoma and fibroblast cell lines was assessed using neutral red uptake (NRU) assay [49]. The cells were plated overnight in 96-well plates (3 × 10^3^ cells/well) and treated with different concentrations of CBD, CBC, CBG, CBN, or 5FU (6.25, 12.5, 25.0, 50.0, and 100.0 µg/mL) or appropriate volume of the solvent control (control cells = 100% viability). Following 48 h of culture, the cells were treated with neutral red (33 µg/mL) for 3 h in the conditioning medium containing FBS (1%). An inverted microscope (Nikon Eclipse, Nikon, Japan) was used to examine the cell morphology, which was documented with an Invenio II camera (DEltaPix, Smørum, Denmark). After DPBS rinsing and lysis with ethanol (50%)+ acetic acid (1%), the absorbance of the neutral red released from the cells was recorded at λ = 540 nm with a FilterMax F5 microplate reader (Molecular Devices, San Jose, CA, USA). The absorbance of the control cells was set as 100% cellular viability and employed to express the percentage of viable cells in the other samples. The concentrations required to decrease the cell viability to 50% (IC_50_) were calculated using a quick fit-dose response with non-linear regression analysis (sigmoidal fit with Boltzmann function).

### 3.4. Melanin Assay

Murine melanoma B16F10 cells were plated onto 6-well plates (1 × 10^5^ cells/well), grown overnight, and treated with 10 nM α-MSH in combination with tested phytocannabinoids (at 2.5 or 5.0 µg/mL) or kojic acid (200 µg/mL) dissolved in cell culture medium. Negative control cells (MSH-) were kept in the culture medium with an equal solvent volume. Following 48 h incubation, the conditioned medium and cell pellets were collected. The cell pellets were dispersed in NaOH (1 N), incubated for 2 h at 80 °C, and centrifuged to remove cell debris. Subsequently, the media and cell lysates were placed in 96-well plates, with the absorbance recorded at λ = 405 nm using the FilterMax F5 microplate reader. The protein content in cell lysates was established by Bradford assay [50] with the DC Protein Assay II kit (Bio-Rad Laboratories, Hercules, CA, USA). The melanin released in the medium and the melanin content in cell lysates (µg melanin/mg protein) were determined using calibration curves with synthetic melanin. The values measured for α-MSH-treated control cells were set as 100% and employed to express the melanin release (extracellular) and melanin content (intracellular) in the samples.

### 3.5. Tyrosinase Activity Assays

#### 3.5.1. Mushroom Tyrosinase Activity Assay

The mushroom tyrosinase activity assay was performed as presented by Uchida et al. [51] with slight changes. Briefly, phosphate buffer (100 mM, pH 6.8, 120 μL), tested compound (final concentration of 50, 100, or 200 µg/mL, 20 µL), and mushroom tyrosinase (500 U/mL, 20 µL) were mixed. After a pre-incubation period of 10 min at room temperature, _L_-DOPA (4 mM, 40 µL) was added. Following an incubation period of 20 min at room temperature in the dark, the absorbance of the formed dopaquinone was recorded at λ = 450 nm using the FilterMax F5 microplate reader. The control sample (100% mushroom tyrosinase activity) comprised phosphate buffer, tyrosinase, L-DOPA, and an equal solvent volume. Kojic acid at comparable concentrations was used as the positive control. The mushroom tyrosinase inhibitory activity was determined as follows: % of mushroom tyrosinase activity = (*A_S_*/*A_C_*) × 100, with *A_S_* representing the sample absorbance (tested compound + tyrosinase + L-DOPA) and *A_C_* representing the control absorbance (solvent + tyrosinase + L-DOPA).

#### 3.5.2. Murine Tyrosinase Activity Assay

Murine tyrosinase activity assay was performed using the lysate of B16F10 murine melanoma cells, prepared as previously described [43]. The B16F10 lysate containing 20 µg protein, tested compound (final concentrations of 50, 100, or 200 µg/mL, 20 µL), L-DOPA (4 mM, 40 µL), and phosphate buffer (100 mM, pH 6.8, up to 200 µL) were mixed. The reaction was conducted in the dark for 4 h at 37 °C. The control sample (100% murine tyrosinase activity) comprised an appropriate solvent volume. Kojic acid at comparable concentrations was used as the control. The dopachrome formation measurement and the murine tyrosinase activity calculation in each sample were obtained as described for the mushroom tyrosinase activity assay. 

### 3.6. Statistical Analysis

The cell-based experiments were carried out in triplicate, with the data representative for at least three individual experiments and presented as mean ± standard error of the mean (S.E.M.). The non-cell-based experiments were conducted in triplicate, with the results provided as mean ± standard deviation (S.D.). The statistical analysis was performed in OriginPro2020 (OriginLab Corp., Northampton, MA, USA) using ANOVA with Tukey’s posthoc test; *p* < 0.05 was considered statistically significant.

## 4. Conclusions

Due to the recently increased interest in cosmetic products containing cannabis or hemp extracts and the growing availability of phytocannabinoid-based cosmetics, there is an imperious need to bring scientific evidence to confirm their safety profile and beneficial effects on the skin. This study depicted the possible involvement of CBD and three minor phytocannabinoids (CBG, CBN, and CBC) in modulating the viability, melanogenesis, and tyrosinase activity of skin cells. Firstly, the four compounds were highly cytotoxic (IC_50_ = 12.02–25.13 μg/mL) to human malignant melanoma A375 cells. Secondly, CBD, CBG, and CBC impaired the melanin synthesis and release in αMSH-stimulated murine melanoma B16F10 cells, as assessed via the reduced extracellular and intracellular melanin content. Lastly, CBG, CBN, and CBC down-regulated the mushroom tyrosinase activity, with CBN additionally inhibiting murine tyrosinase. Thus, by evaluating for the first time the preliminary anti-melanoma, anti-melanogenic, and anti-tyrosinase properties of CBN and CBC and confirming similar effects for CBD and CBG, this study can expand the utilization of CBD and, in particular, of minor phytocannabinoids to novel cosmeceutical products for skin care. However, further investigations are needed to elucidate their target receptors or signaling pathways, as well as to extend the phytocannabinoid research to animal or human studies. 

## Figures and Tables

**Table 1:** IC_50_ values of phytocannabinoids in malignant melanoma cells.

Cell Line	CBD	CBG	CBN	CBC	5FU
	IC_50_ [μg/mL]				
A375	12.02 ± 0.60	12.14 ± 2.34	25.13 ± 1.68	23.00 ± 2.60	29.96 ± 0.78
SH4	>100	>100	>100	>100	>100
G361	>100	>100	>100	>100	>100
BJ	>100	>100	>100	>100	>100

5FU, 5-fluorouracil, CBC, cannabichromene; CBD, cannabidiol; CBG, cannabigerol; CBN, cannabinol.

## Data Availability

All data are contained within the manuscript.
